# Optical trapping stability of different irregularly shaped microplastic particles

**DOI:** 10.1038/s41598-025-02571-1

**Published:** 2025-05-22

**Authors:** Noorulhoda Kazemi, Mimi Truong, Alexander B. Stilgoe, Viktor Nascak, Jesus Poblano, Anna Bezryadina

**Affiliations:** 1 Department of Physics and Astronomy, California State University Northridge, Northridge, CA 91330 USA; 2 Department of Computer Science, California State University Northridge, Northridge, CA 91330 USA; 3 School of Mathematics and Physics, The University of Queensland, St. Lucia, Brisbane, QLD 4072 Australia; 4 ARC Centre of Excellence for Quantum Biotechnology, School of Mathematics and Physics, The University of Queensland, St. Lucia, Brisbane, QLD 4072 Australia

**Keywords:** Optical tweezers, Microplastics, Asymmetric particle, Raman spectroscopy, Optical manipulation and tweezers, Raman spectroscopy

## Abstract

Plastic pollution has become a major environmental issue. Waste degrades into microplastics and nanoplastics, which contaminate water, soil, and air, and affect ecosystems and food sources. To elucidate the effects of microplastics on cellular systems, it is essential to comprehend their properties and manipulation at the microscopic scale. This work examines the optical trapping stability of different irregularly shaped laboratory-synthesized, mechanically weathered microplastics: polypropylene (PP), polyethylene terephthalate (PET), and high-density polyethylene (HDPE). We conducted a statistical assessment of optical trapping stability, considering factors such as particle material, color-induced absorption, size, and response to different optical trapping wavelengths (473 nm, 780 nm, and 820 nm). Additionally, we compared these results with the predicted optical trapping stability, simulated for particles with two types of spheroidal shapes. Our results indicate that non-spherical PP microplastics exhibit the highest stability in a single-beam optical trap, while PET microplastics demonstrate the lowest stability. The optical trapping stability of PP and HDPE microplastics is relatively size-independent; however, PET particles larger than 10 μm are three times less likely to be stably trapped than smaller particles. Furthermore, non-transparent materials with higher absorption rates cause less stable optical trapping of microplastics for all three material types. The insights gained regarding the optical properties of irregularly shaped microplastics will help future research on the optically controlled interactions of naturally occurring microplastics with cells and microorganisms at the single-cell level.

## Introduction

The discovery of plastic made it possible to produce goods and household products made from materials that are inexpensive, durable, corrosion-resistant, electrically insulating, and convenient to use and process^1–4^. Food and drink containers made of more costly glass and aluminum were swiftly supplanted by plastic packaging. Nowadays, 40% of all plastic materials are used to stock and package products from various manufacturers across the globe^5^. Due to the cheap and easy production of plastics, the use of plastics has experienced exponential growth, with the worldwide production of plastic reaching almost 400 million tons per year in 2021^6^. Unfortunately, despite being recyclable materials, only about 9% of generated plastic waste has been recycled, 12% has been incinerated, and the rest has accumulated in landfills and the environment^5,7^. If the current trend in the production and consumption of plastics continues, by 2050, more than 1.2 billion tons of plastic waste are projected to accumulate in the environment^7^.

Due to plastic’s durability and resistance to many chemicals, plastic degradation takes between 20 and 1000 years depending on the type and thickness of plastic and environmental conditions^8^. When exposed to sunlight and various chemical and physical stressors, billions of tons of discarded plastic waste are gradually decomposed into fragments and then degraded into microplastics (5 mm to 1 μm in size) and nanoplastics (less than 1 μm in size)^3,9^. Since microplastics are small in size, they are easily dispersed resulting in contamination of water, soil, and air^9–14^. Microplastic particles have been detected in drinking water, food, animals, plants, and humans^6,15,16^. Microplastics and nanoplastics are easily ingested by microorganisms and later digested by larger species and humans. Microplastics can enter the human body through the digestive system, inhalation, and even direct skin contact^14,17–19^. Multiple studies have shown that microplastic with a size as large as 10 μm can pass through the epithelium in vitro and enter organs^9,20,21^. It is important to develop methods to manipulate microplastic particles to enable studies of their impact on cells and microorganisms at the microscopic level. With a well-tuned microscope and a simple optical trapping system, microplastics can be transported to study interactions with cells.

Since Ashkin’s early work, optical trapping has become a common tool to manipulate objects of different sizes and shapes in suspension^22–27^. The optical gradient force from a highly focused laser beam attracts a particle toward the center of the beam, allowing the user to hold and manipulate the particle with high precision. The particle stability in the optical trap is determined by laser light and particle properties (such as laser wavelength and power, quality of the laser beam profile, size of the focal spot, particle size, shape, transparency, density, and index of refraction), as well as optical trapping depth from the objective and suspension media selection. Optical trapping of spherical particles of different sizes, indices of refraction, and transparency is extensively studied experimentally and theoretically. Theoretical models have been developed to describe the optical trapping behavior of spherical particles with different sizes and indices of refraction^25,26,28^. Recent computational methods have allowed the calculation of the optical forces of some non-spherical symmetric particles^29–32^. Several studies have shown that optical trapping and manipulation of large, irregularly shaped particles are possible^33,34^; however, much remains unclear about the trapping behavior of asymmetric and irregular particles.

In this work, we investigate how well the spheroidal approximation can predict the behavior of a strongly asymmetric particle in a single Gaussian optical trap and when the spherical approximation starts to fail. We present experimental data and statistical assessments of the optical trapping stability of various microplastic materials (polypropylene (PP), polyethylene terephthalate (PET), and high-density polyethylene (HDPE), colors, and sizes, as well as responses at different optical trapping wavelengths (473 nm, 780 nm, and 820 nm). Weathered microplastics found in nature have large variations in size and possess generally irregular and asymmetric shapes. We investigate the optical trapping properties of irregularly shaped microplastics ranging in size from 1 μm to 50 μm. In addition, we compare our experimental results to the expected model of optical trapping stability, which is calculated for spheroidal particles in the optical trap for different indices of refraction, sizes, and ellipsoidal shape orientation. The theoretical model accounts for Gaussian beam aberration, which is unavoidable when trapping larger particles farther away from the sample’s cover glass surface. Both theoretical and experimental results show that all PP particles are fairly robust in an optical trap, but large PET particles are significantly less likely to be trapped and held. The results show that irregularly shaped particles have a similar trend in optical trapping stability to spherical particles and depend on the index of refraction and optical absorption. However, the particle density and index of refraction play a significantly larger role in particle stability of large irregularly shaped particles (geometric regime) than what a spheroidal approximation predicts.

**Fig. 1:**
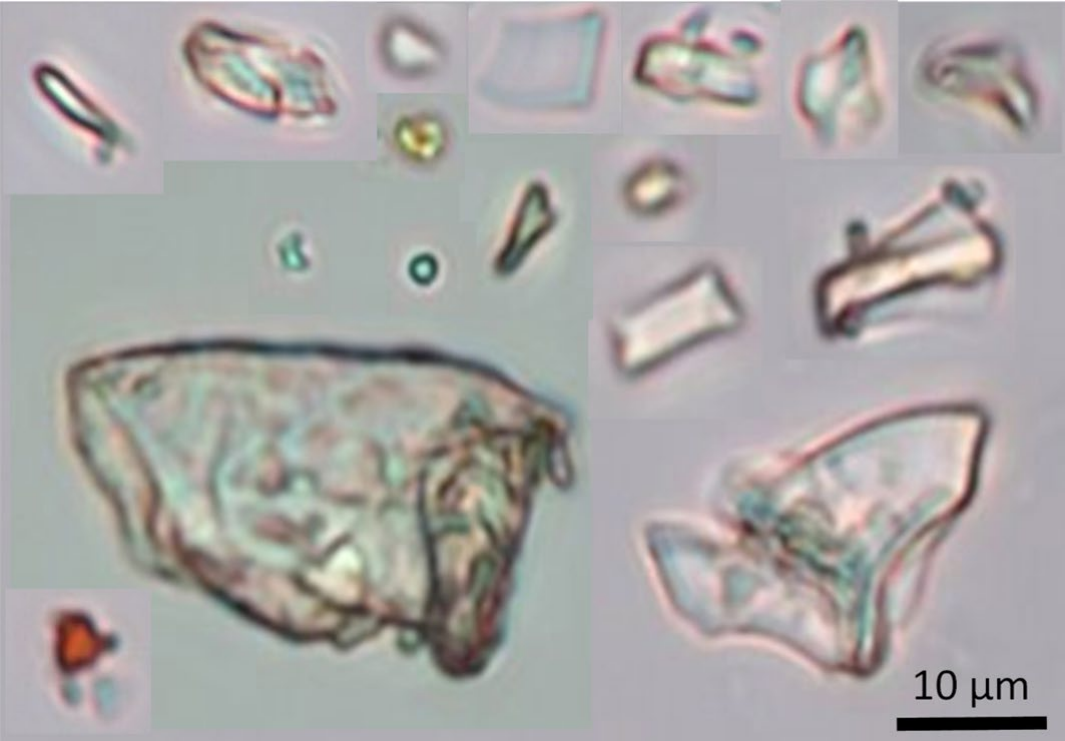
Microplastics found in the ocean with different sizes and irregular shapes.

## Results

### Irregularly shaped microplastic particles and their properties

**Fig. 2:**
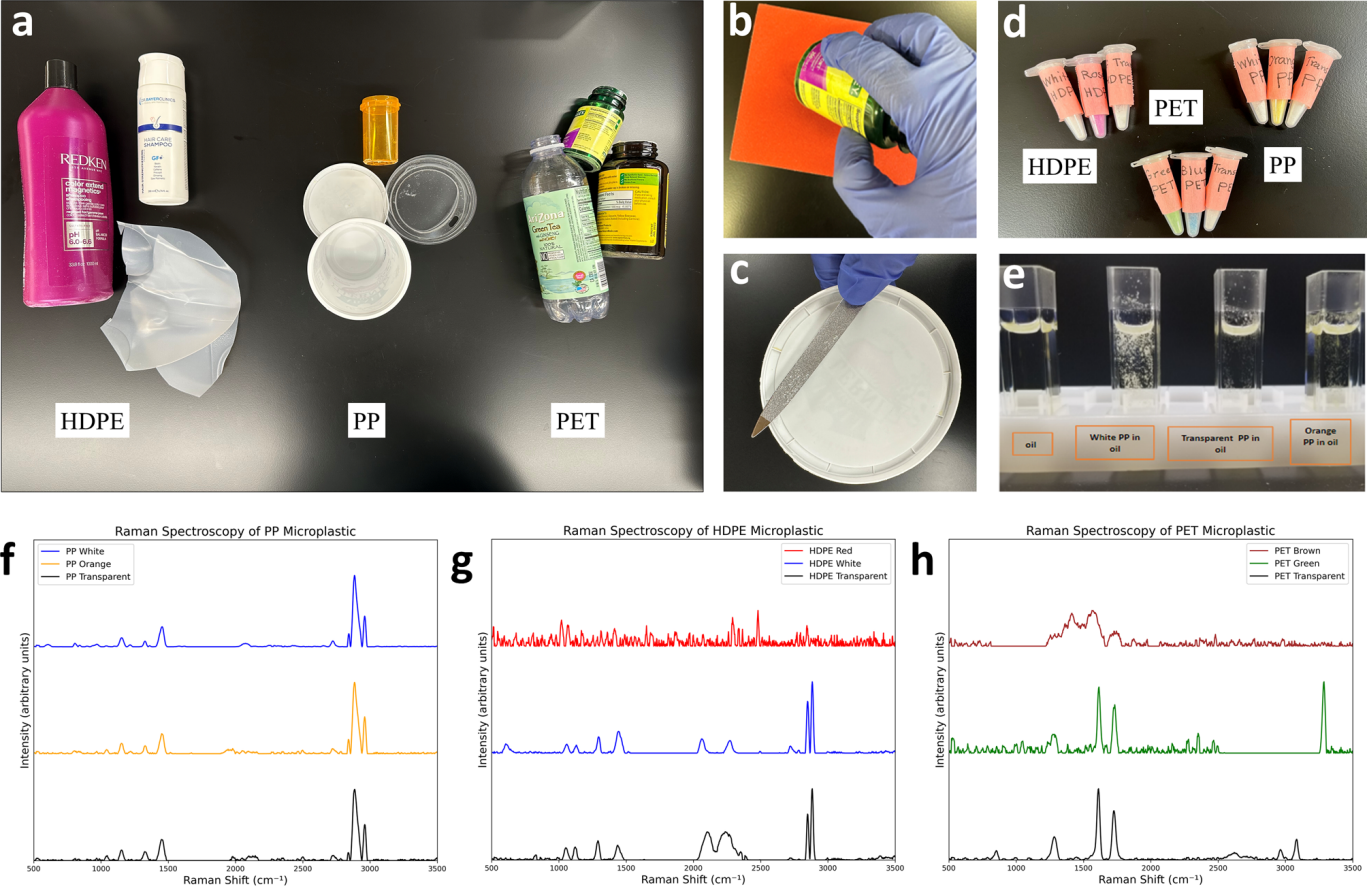
Preparation of lab-made microplastics. (**a**) Collection of different types of plastic waste; (**b**) Sanding down microplastics with sandpaper. (**c**) Preparation of microplastic samples with individual stainless-steel files. (**d**) Tubes with lab-made HDPE, PET, and PP microplastics; (**e**) Microplastics suspended in soybean oil for absorption measurements. (**f**–**h**) Raman spectra of PP, HDPE, and PET microplastic samples.

Microplastics in the ocean often have irregular shapes, making them difficult to distinguish from sand (Fig. 1). Raman tweezers are used to identify microplastics based on their molecular composition, but applying this technique consistently can be challenging due to factors like particle fluorescence and surface heterogeneity, which can interfere with clear identification^34–36^. To systematically investigate the optical characteristics of irregularly shaped microplastics, lab-made microplastics were created by collecting and mechanically weathering household items like shampoo bottles, food containers, and pill bottles (Fig. 2a). The study focused on the three most common plastics present in the environment: polypropylene (PP), polyethylene terephthalate (PET), and high-density polyethylene (HDPE). Polypropylene (PP) and polyethylene (PE) account for more than half of all used plastics, and PET is the most common thermoplastic used for water bottles and packaging^4,7^. Table 1 includes relevant information regarding the three selected types of plastics^37^.

**Table 1:** Different types of plastics and their properties.

Name of Material	Refractive Index	Density (g/cm^3^)	Water Absorption over 24 h
Polypropylene (PP)	1.46	0.85–0.90	Unaffected by water
High-density polyethylene (HDPE)	1.54	0.95	< 0.01
Polyethylene Terephthalate (PET)	1.58–1.64	1.3–1.4	0.1

All three materials have a high index of refraction, resulting in microparticles with positive polarizability that are attracted to the optical trap^28^. PP and HDPE materials have a lower density than water, whereas PET materials have a higher density, causing lab-made microplastics to gently rise or sink in water over time. PET is a hygroscopic thermoplastic that absorbs moisture from its surroundings, which can lead to changes in PET microplastic characteristics over time. To minimize the effects of both water absorption and the potential release of additives or degradation products into the surrounding medium, fresh particle suspensions were prepared daily, and all measurements were conducted shortly after preparation. Finally, to analyze the impact of microplastic color and transparency on optical properties, we prepared microplastic samples from plastic trash of different colors and transparency levels.

The initial phase of plastic weathering in nature is often mechanical, so we chose the mechanical method to create our samples. We sanded down selected plastic items with fine sandpaper or metallic files to produce lab-made microplastic samples (Fig. 2(b-d)). For optical tweezers experiments, we study the optical effects of individual particles, and individual metallic files are employed. For absorption measurements, a large amount of sample is needed and fine sandpaper is a better choice to create a microplastic sample. To minimize the floating of microplastics during absorption measurements, they are suspended in soybean oil instead of water (Fig. 2(e)). (See Methods for sample preparation details.)

The same type of plastic waste often varies in properties due to factors such as impure composition, dyes or pigments, different polymeric lengths, or cross-linking structures. To identify the polymers, we performed Raman spectroscopy with 532 nm laser excitation on plastic materials to determine their chemical composition (Fig. 2(f-h)). We compared the observed Raman spectra with the main Raman vibrations of plastic polymers^38,39^.

The Raman peaks for PP microplastic occur around 1045 cm^− 1^ (C-CH_3_ stretching, C-C stretching, and CH bending), 1153 cm^− 1^ (C–C and C–CH_3_ stretching, CH bending and CH_3_ rocking), 1327 cm^− 1^ (CH bending and CH_2_ twisting), 1452 cm^− 1^ (asymmetric bending of CH_3_ and CH_2_ bending), 2840 cm^− 1^ (CH_2_ stretching), 2884 cm^− 1^(symmetric stretching of CH_3_), and 2960 cm^− 1^ (asymmetric stretching of CH_2_).

For HDPE microplastic, the Raman peaks are found around 1054 cm^− 1^ (C-C asymmetric stretching), 1125 cm^− 1^ (C-C symmetric stretching), 1293 cm^− 1^ (CH_2_ twisting), 1440 cm^− 1^ (CH_2_ deformation), 2849 cm^− 1^ (symmetric CH_2_ stretching), and 2883 cm^− 1^ (asymmetric CH_2_ stretching). HDPE microplastic also exhibits additional broad peaks around 2080 cm^− 1^ and 2250 cm^− 1^, which are likely attributed to additives or degradation, corresponding to C ≡ N stretching vibrations.

The Raman characteristic peaks for PET microplastic occur around 1283 cm^− 1^ (C-C stretching (ring), C-O stretching), 1613 cm^− 1^ (C = C stretching), and 1729 cm^− 1^ (C = O stretching). Additional clear peaks for transparent PET microplastic are observed at 851 cm^− 1^ (C-C stretching (ring breathing), C-O stretching), 2962 cm^− 1^ (CH_2_ stretching), and 3084 cm^− 1^ (CH stretching). For green PET microplastic, there is an additional peak at 3287 cm^− 1^, likely due to additives, contaminants, or adsorbed moisture.

The transparent microplastic samples exhibited clear Raman spectra corresponding to all three types of materials. However, identifying certain colored plastic samples proved more challenging, and in some cases, it was not possible^35,36,40^. Additive compounds, particularly color additives, can partially or completely obscure the Raman spectra of the material^39^. Raman spectra of our brown PET and red HDPE samples could not be collected with 532 nm laser excitation at all. Previous studies have shown that red pigmentation often obscures polymer identification due to peak broadening and fluorescence effects^36,40^. The dyeing process of brown PET and red HDPE may introduce fluorescent substances, such as whitening agents and dyes, which alter the Raman spectra of the microplastics. This results in a strong fluorescence background that masks the characteristic polymer peaks.

Due to the high background noise and fluorescence observed in several samples, detailed identification of additives, dyes, or potential leachates was not possible. To minimize variability related to chemical composition, samples from three different household sources were analyzed for each type of plastic. To overcome this challenge, higher-sensitivity Raman spectroscopy with multiple excitation lasers, or complementary chemical analyses such as Surface-Enhanced Raman Spectroscopy (SERS) will be needed to improve spectral resolution, enabling more accurate additive identification, and better assessment of their potential influence on optical trapping behavior.

**Fig. 3:**
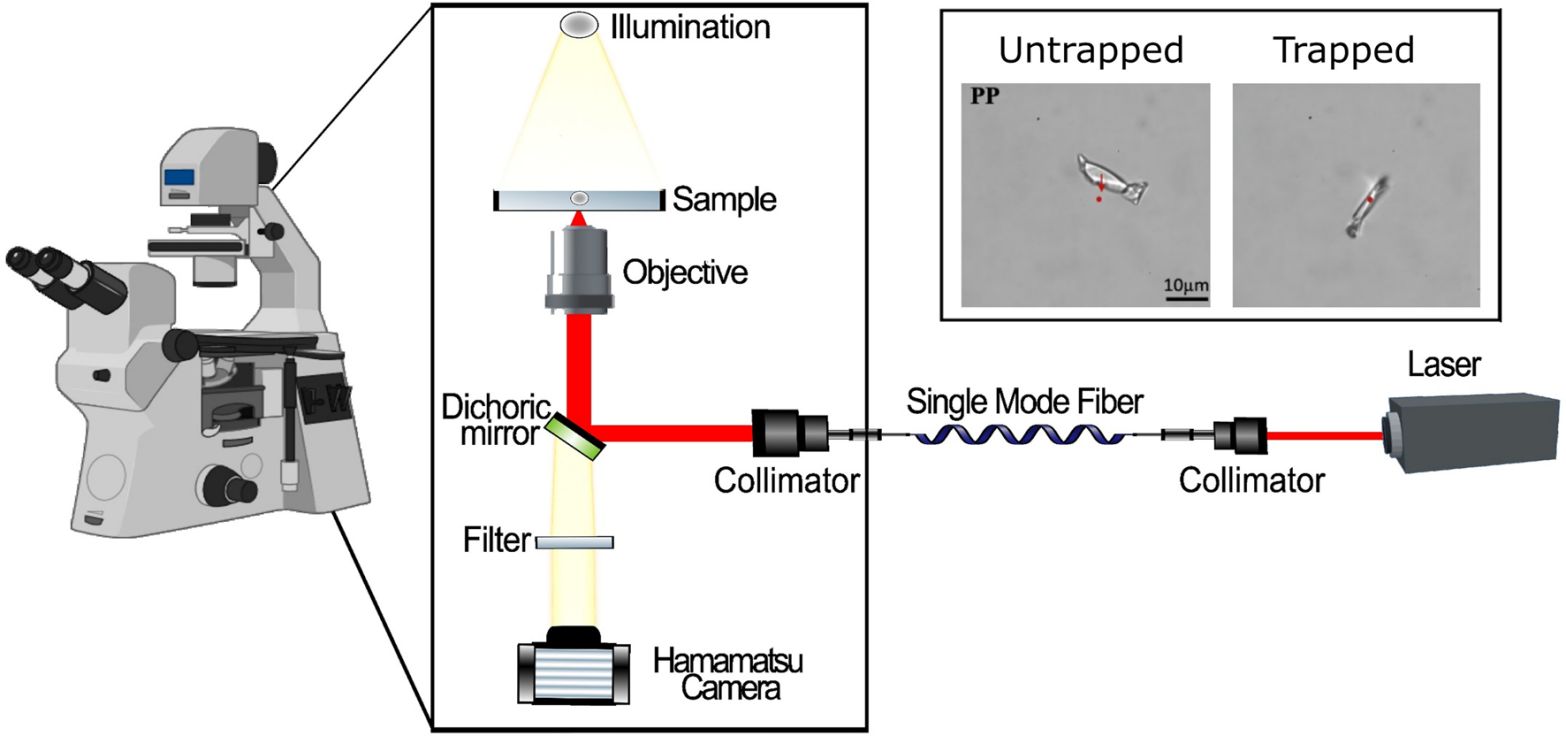
Schematic illustration of the experimental setup. The laser beam is sent via a fiber into the inverted microscope and focused by the high numerical aperture objective onto the substrate. (Inset) Optical trapping of PP microplastic when laser light is turned on.

### Measurements of optical trapping stability of microplastics

The setup we used to study the optical trapping stability of microplastics is illustrated in Fig. 3. The single-trap optical tweezers system is built around an inverted microscope with a laser beam delivered through a single-mode optical fiber (see Methods for setup details).

**Fig. 4:**
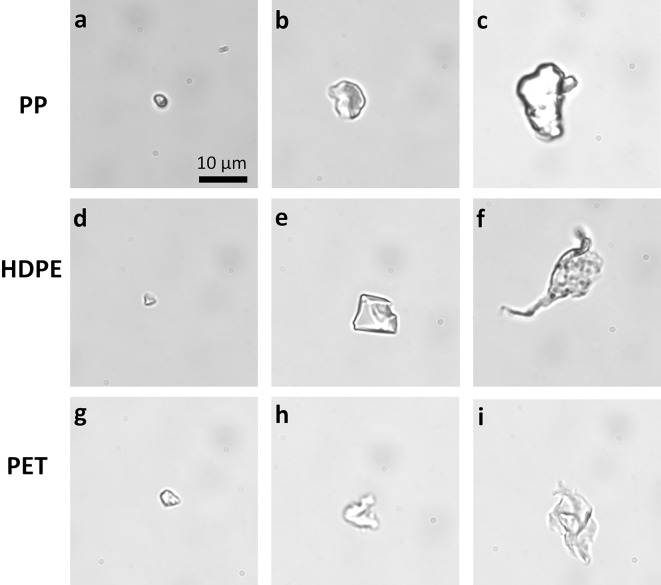
Different sizes and shapes of lab-made microplastic particles: (**a**–**c**) PP microplastics; (**d**−**f**) HDPE microplastics; (**g**–**i**) PET microplastics.

For each material type and color of microplastics (9 cases), we examined the optical trapping behavior of at least 20 particles of varying sizes for each optical trapping wavelength (820 nm, 780 nm, and 473 nm). These wavelengths were selected to test two distinct regions of the optical spectrum — visible and near-infrared — in order to investigate potential wavelength-dependent effects on optical trapping behavior. The 473 nm laser, representing the visible range, is more likely to cause absorption and heating effects, while the 780 nm and 820 nm near-infrared lasers are commonly used in biological studies due to their lower absorption in aqueous environments. Figure 4 illustrates microplastics of different sizes and shapes. The particle sizes ranged between 1 μm and 50 μm, with most particles falling between 3 μm and 20 μm. Optical trapping tests were performed using a 1.25 NA 100x objective, with particles trapped at an approximate depth of 10–50 μm from the cover glass. In total, 540 experimental measurements were used to assess the optical trapping stability of the particles based on their properties. To ensure representative sampling and minimize sampling bias, particles were selected for trapping experiments as they appeared in the observation region. To ensure size distribution in each set of experiments, roughly half of the samples were large (more than 10 μm in length) and the other half small (less than 10 μm in length). Since size measurements were done after the experiments, the exact distribution varies. The full set of collected data is provided in Table 2. A particle is considered stably trapped if it can be optically manipulated across the sample by moving the microscope stage. To calculate the error bars for the collected data, we used the confidence limit method for probability events^41^. The data from Table 2, along with error bars, is presented in subsequent graphs that compare the effects of various microplastics’ properties.

**Table 2:** Optical trapping stability of different microplastics based on type, size, color, and optical tweezers wavelength. N_st_ is the number of stably trapped particles for each set of conditions. N_unst_ is the number of unstably trapped particles for each set of conditions.

Wavelength		473 nm			780 nm			820 nm		
Type	Color/ Size	*N*_st_	*N*_unst_	Stability %	*N*_st_	*N*_unst_	Stability %	*N*_st_	*N*_unst_	Stability %
PP	White	15	5	75	17	3	85	17	3	85
	Transparent Orange	16	4	80	18	2	90	18	2	90
	Transparent	17	3	85	18	2	90	18	2	90
	Small	26	6	81	30	2	94	31	7	82
	Large	22	6	79	23	5	82	22	0	100
	All Types	48	12	80	53	7	88	53	7	88
HDPE	White	10	10	50	8	12	40	10	10	50
	Red	11	9	55	12	8	60	12	8	60
	Half Transparent	12	8	60	16	4	80	13	7	65
	Small	12	17	41	27	16	63	18	15	55
	Large	21	10	68	9	8	53	17	10	63
	All Types	33	27	55	36	24	60	35	25	58
PET	Green	5	15	25	4	16	20	7	13	35
	Brown	7	13	35	6	14	30	11	9	55
	Transparent	8	12	40	8	12	40	12	8	60
	Small	16	17	48	6	2	75	27	11	71
	Large	4	23	15	12	40	23	3	19	14
	All types	20	40	33	18	42	30	30	30	50

### Stability of optical trapping based on material and laser wavelength

All three types of microplastics are attracted to the optical trap for all three wavelengths. Nonetheless, some particles exhibit greater stability than others inside the optical trap. When optical trapping forces are large and evenly distributed around a particle, particles of all sizes and materials are trapped stationary. Supplementary Videos S1 and S2 show examples of stable optical trapping of PP and PET particles, respectively. In these videos, a particle is placed near the position of a future optical trap, and when the laser light is turned on, the particle is attracted toward the laser. After the particle is trapped, the microscope stage is moved while the particle remains in the trap. This method allows us to check whether the optical forces can hold particles securely and endure viscous drag forces as well as minor obstructions in the captured particle’s path.

Even if a particle is asymmetric, it is possible to trap some particles. However, if the optical forces are unevenly distributed over the trapped particle, the asymmetric particle experiences optically induced torque, causing it to spin about its axis. This is the most common case for irregularly shaped microplastic particles. Supplementary Videos S3, S4, and S5 are examples of optically trapped HDPE, PP, and PET particles that spin and can be moved around with optical tweezers. We consider these particles to be stably trapped in our statistical analysis because they can be optically manipulated across the sample.

If optical trapping forces are weak for some particle materials, shapes, and laser wavelengths, asymmetry can cause particles to be pushed out of the trap. Supplementary Video S6 shows an asymmetric PET particle being optically trapped and spinning before being forced out of the trap after a few rotations. Supplementary Videos S7, S8, and S9 show examples of unstable optical trapping of HDPE, PET, and PP particles, respectively, in which particles cannot be trapped even for a short duration. In these videos, when the laser beam is turned on, the particle is initially attracted and then immediately pushed out of the beam. The videos show multiple attempts to trap the same particle by bringing it closer to the location of the optical trap and then turning on the laser beam. It is also worth noting that the optical trapping forces and particle stability within the trap are significantly influenced by the trapping depth. The effect is particularly noticeable with the 473 nm laser. To ensure consistent results, particles were trapped near the sample’s cover glass at a depth of no more than 100 μm. Most measurements were taken at a depth of 10–50 μm from the cover glass. However, the exact depth at each experimental measurement is difficult to determine since the focus position slightly drifts over time and the focus position has to be tweaked to find microplastic particles in samples.

**Fig. 5:**
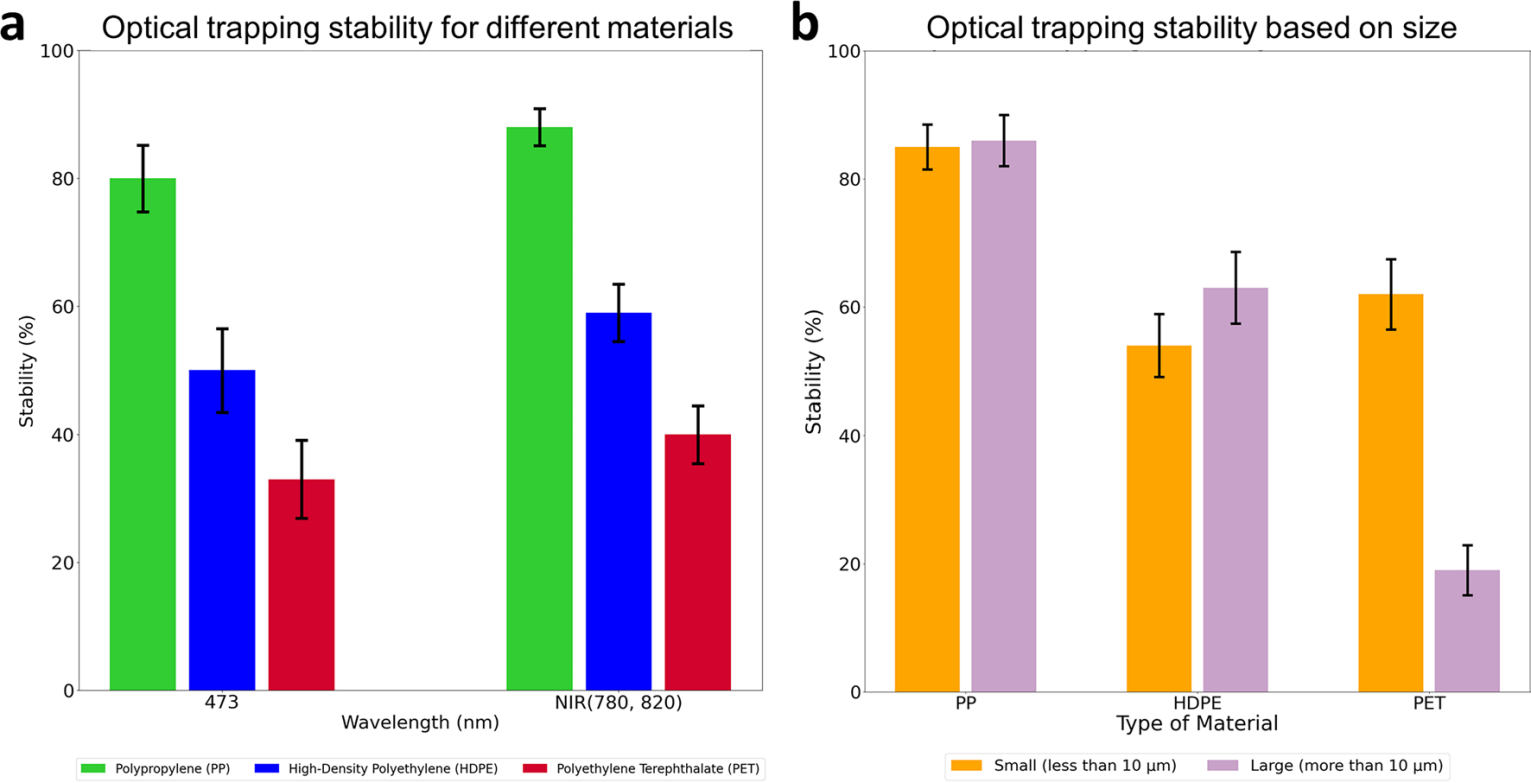
Comparison of optical trapping stability based on type and size of microplastics and laser wavelength. The error bars are calculated using the confidence limit method for probability events. (**a**) Optical trapping stability of microplastic for different materials (PP, HDPE, and PET) at 473 nm and low NIR (780 nm and 820 nm) laser wavelengths. (**b**) Optical trapping stability based on the size of microplastics: less than 10 μm and larger than 10 μm in length.

Figure 5 (a) illustrates the stability results of microplastics based on material type, using both 473 nm and low NIR (780 nm and 820 nm) laser wavelengths. PP exhibits the highest stability, followed by HDPE, and PET demonstrates the lowest stability. The histogram aggregates measurements from 60 different particles for each wavelength and material, independent of particle size or color. The observed differences in stability between materials are predominantly due to differences in the index of refraction of the particles, with PP having an index of 1.46, HDPE around 1.54, and PET above 1.56.

The stability patterns for different materials are similar for both low NIR and blue lasers. The main difference is that, under the blue laser, particles exhibit slightly reduced stability due to heating effects. Specifically, with the blue laser (473 nm), all material types show some degree of absorption. The heating effects on microplastics, particularly for non-transparent ones, can lead to bubble formation from vaporized water or changes in the optical trapping properties of microplastic particles over time (more than 20 min). In contrast, with the NIR laser (780 nm and 820 nm) no significant heating effects on microplastics were observed, suggesting that prolonged optical trapping may be possible in future biological studies.

### Stability of optical trapping based on size and shape

The most unexpected result was the optical trapping stability of irregularly shaped microplastics based on particle size (Fig. 5b). Particles were classified as “large” if their length exceeded 10 μm and as “small” if their length was less than 10 μm. The 10 μm threshold was selected because particles larger than 10 μm behave as geometric particles for the most common optical trapping wavelengths. Additionally, previous studies on the toxicity of microplastics have set 10 μm as the upper limit for intracellular uptake^20,21^. For the histogram, the number of analyzed samples for each material varies depending on the size of the microplastics, as the exact size of the observed particles was determined after the observations. Table 2 contains the size distribution of the particles. Data analysis indicates that PET particles larger than 10 μm are three times less stable in optical traps than smaller PET particles. Videos showing the optical trapping behavior of PET particles can be seen in Supplementary Videos S2, S5, S6, and S8. In contrast, PP and HDPE particles are less dependent on particle size. Videos showing optical trapping of various sizes of PP and HDPE particles can be found in Supplementary Videos S1, S3, S4, S7, and S9. The difference in the stability of PET microplastics based on size is most likely due to particle density and its higher index of refraction. PET has a higher density than water, whereas PP and HDPE have a lower density, which affects the particles’ buoyancy and the viscous drag forces acting on large, asymmetric particles. These asymmetric particles are large enough that buoyancy also influences their ability to move and spin due to optically induced torque. Additionally, PET’s higher index of refraction increases scattering forces, which reduces optical trapping stability in the axial direction. The larger size and inhomogeneous structure of the particles further enhance scattering forces and reduce stability.

Since microplastic particles are created by sanding plastic material with a metal file, the shape of irregular particles varies from more symmetrical elliptical-like particles to completely asymmetric thin, flat particles (see Fig. 4). However, since all created particles are irregularly shaped and unique, it is impossible to precisely categorize them into distinct sub-categories based on their shape. Nevertheless, we can firmly conclude that for all samples and laser wavelengths, the asymmetric flat particles are less stable than more symmetric particles. The asymmetric particles are more often attracted to the optical trap and then immediately pushed away, which results from asymmetric optical force distribution across a particle inside a symmetrical optical trap. As a result, the least stable particles are flat, asymmetric, large, non-transparent PET particles, and the easiest to capture and hold particles are symmetrical, transparent PP particles.

### Stability of optical trapping based on color and absorption

**Fig. 6:**
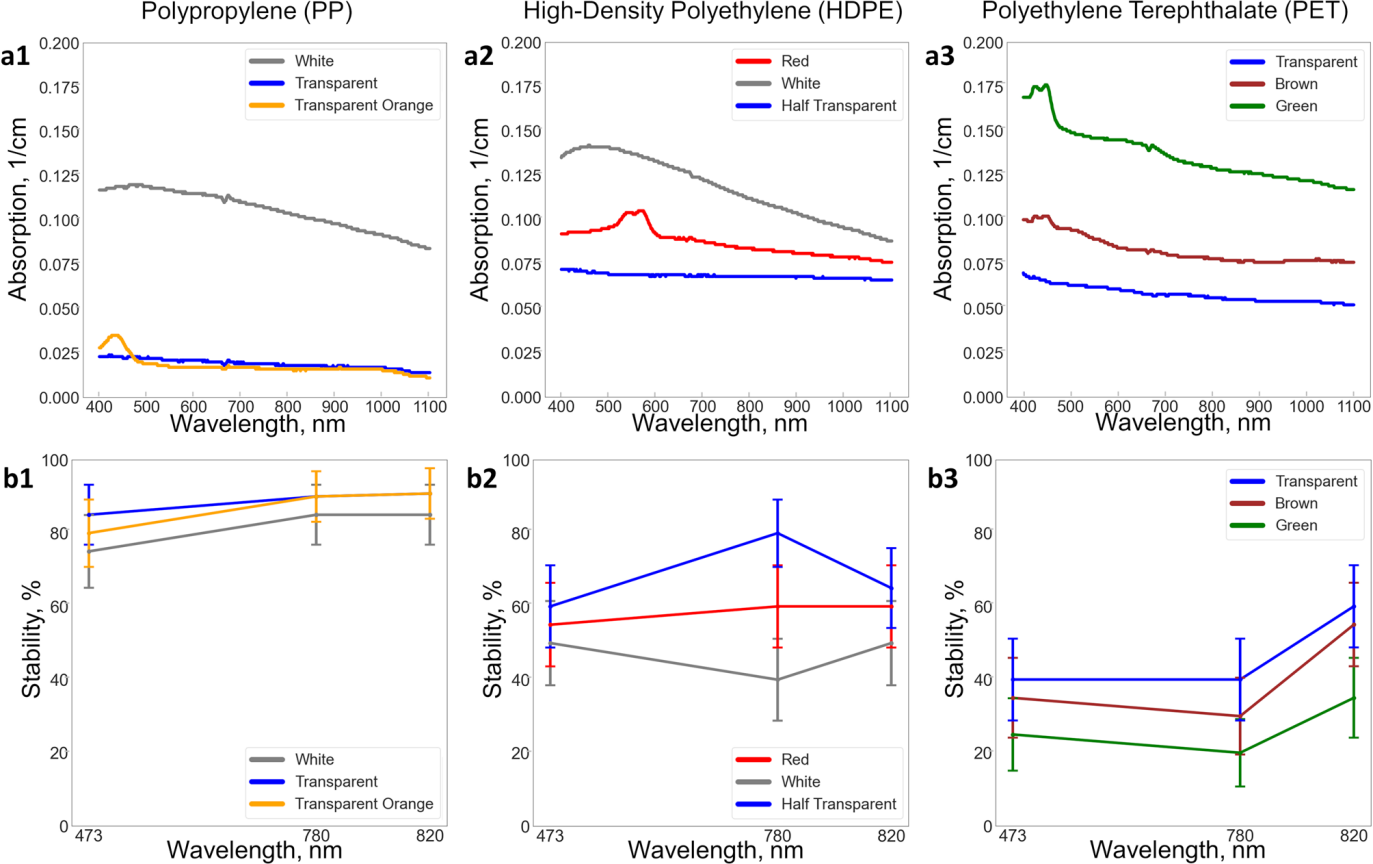
Absorption and optical trapping stability. (**a1**–**a3**) Absorption of microplastics (PP, HDPE, and PET) suspended in soybean oil with respect to oil baseline. (**b1**–**b3**) Optical trapping stability of microplastics (PP, HDPE, and PET) depending on particles’ color and transparency.

To better assess the dependence of optical stability on absorption, we needed to measure the absorption values of particle suspensions rather than bulk media of the same material. This is important since the optical properties of micro-sized particles may differ from those of the bulk material. To determine the absorption of lab-made microplastics using a spectrophotometer, the microplastics were suspended in soybean oil rather than water to prevent particles from floating on the solution’s surface. Figure 6(a1-a3) illustrates the absorption spectra of PP, HDPE, and PET microplastics. Since the spectrophotometer measures the transmission through the sample, the absorption graphs also account for scattering effects in the suspension, which depend on the refractive index. While the spectrophotometer’s absorption graphs are not perfect, they offer a relative comparison between different colors of materials of the same type. For all three types of microplastics, the absorption of transparent samples is lower than that of colored samples. Surprisingly, the color of plastic does not result in high absorption peaks. PP microplastics have lower absorption spectra compared to HDPE and PET microplastics, especially for the transparent PP microplastics.

When analyzing optical trapping stability based on material color and transparency, it becomes clear that the absorption and transparency of the plastic strongly influence trapping stability. A higher absorption rate leads to less stable optical trapping of microplastics. Figure 6 (b1-b3) shows the optical trapping stability of PP, HDPE, and PET microplastics of different colors and transparency using 473 nm, 780 nm, and 820 nm lasers. For these plots, we used the optical trapping stability data of 20 particles for each color of microplastic. Lower absorption materials provide better optical trapping stability across all microplastics and wavelengths. Since the experiments were conducted with the same laser wavelength for all samples before transitioning to another wavelength, the results showed a slight variation in optical trapping stability between the 780 nm and 820 nm datasets. These variations, however, fall within the error bars. Overall, we can conclude that the optical trapping stability of irregularly shaped particles based on wavelength and material absorbance is consistent with the general understanding of optical trapping stability for spherical particles.

## Discussion

A certain amount of spherical aberration affected trap quality in the experiments. Oil-water interfaces, such as those present in our experiments, produce progressive spherical aberration with increasing focal depth. In imaging, increased spherical aberration leads to reduced resolution. For optical trapping, depth-related beam quality reduction can prevent stable trapping. Our calculations of optical trapping combine two computational packages^32,42^ that enable electromagnetic modeling of optical forces and torques influencing particles from the sub-wavelength to the 10-micron size scale. Electromagnetic theory is more appropriate than ray-based models^43,44^ in this study, as it accounts for aberration, interference, and size effects.

Non-spherical particles experience optical forces that depend on both their orientation and position. Simulations of light-scattering spheroids were conducted to assess the dynamics of particles with different sizes, shapes, and orientations. As the simplest non-spherical particles, they are an ideal way to gain insight into the behavior of irregularly shaped microplastics using a few representative examples. Two sets of sizes were considered for two types of shapes: prolate spheroids (needle-like particles) and oblate spheroids (disk-like particles). Table 3 summarizes the simulation parameters used and the range of trap stability. A particle is considered partially stable when it does not remain entirely within the “restoring force” region, and is considered stable when the outer ranges of calculated optical force become negative.

**Fig. 7:**
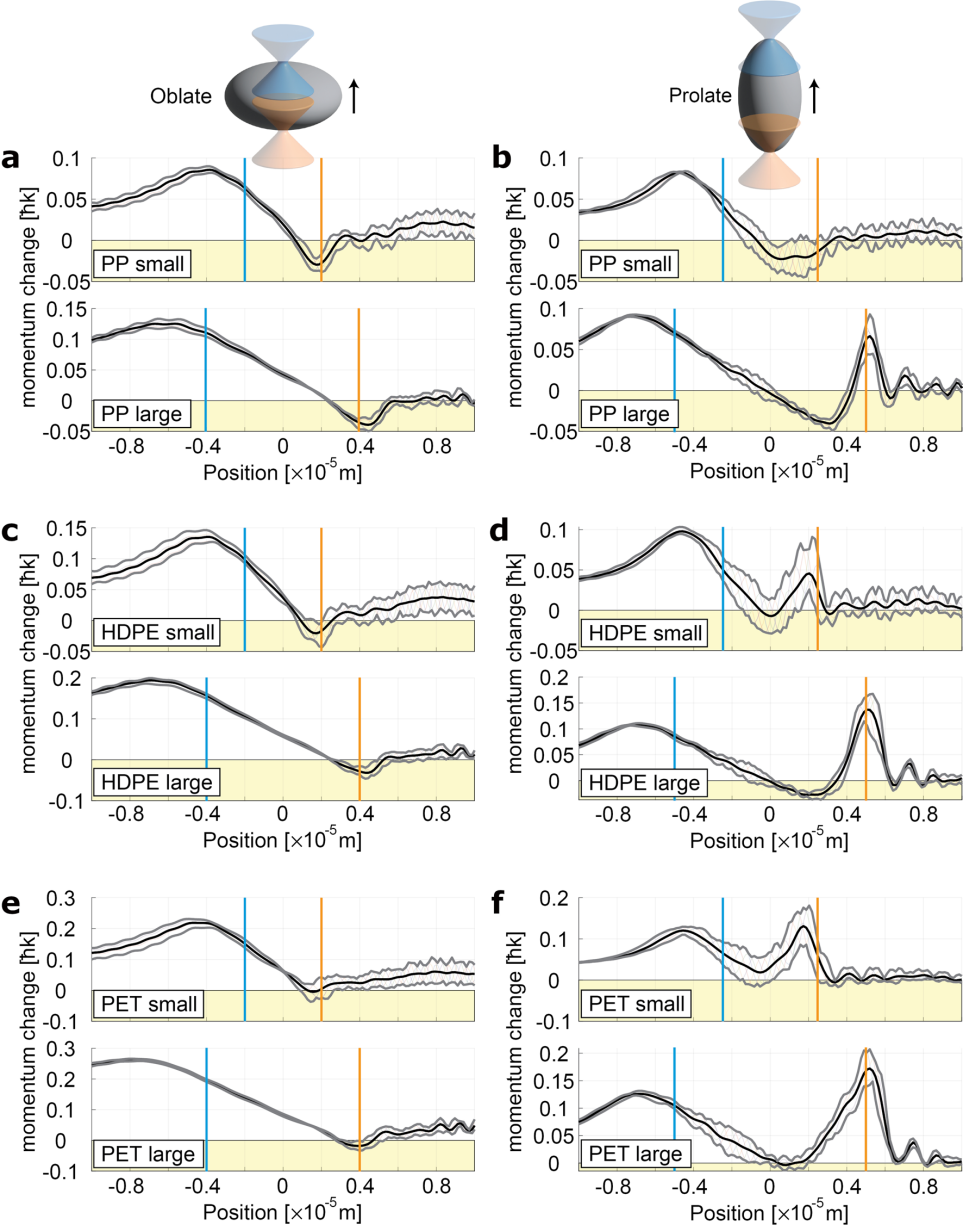
Momentum transfer per light quantum (momentum change, ℏk) to oblate and prolate particles in an aberrated, highly focused laser. The blue line represents the particle position as it moves into the focus along the beam propagation direction (up the page). The orange line represents the point where the particle leaves the focal region of the beam. (**a**–**f**) Subplots correspond to combinations of particle shape (column: oblate/prolate) and material (row: refractive index). The yellow shaded area represents where optical forces move the particle toward the beam source.

Figure 7 shows the momentum transfer per light quantum (momentum change, ℏk, where k is the wavenumber) to the particle as a function of particle position in the beam model^45^. The colored vertical lines represent the position of the aberrated beam’s focus on each edge of the particle. Note that because aberrations distort the beam’s focus, the term “focus” is used in relation to the beam model. The blue line represents the particle position as it moves into the focus along the beam propagation direction. The orange line indicates the point where the particle leaves the focal region of the beam. Spherical aberration results in highly variable trapping behavior for particles of different shapes. To simulate natural variation in a population of particles and account for the effects of interference, the calculations were performed for particles ranging from 0.9 to 1.1 times the particle sizes defined in Table 3. This enables an evaluation of the impact of slight fluctuations in momentum transfer to the particles simulated in Fig. 7 and assess their optical trapping stability. For small particles, the x-coordinate of each force profile corresponds to the true position. For large particles, where the geometric optics regime is valid, the distance has been normalized, and the x-coordinate represents the true position for average-sized particles.

**Table 3:** Simulation parameters and result observations.

Figure 7 part	Average size (µm)			Refractive index	Observation
	width	Depth	height		
a	4	4	2	1.46	Stable
	8	8	4		Stable
b	2.5	2.5	5		Stable
	5	5	10		Stable
c	4	4	2	1.54	Stable
	8	8	4		Stable
d	2.5	2.5	5		Partially Stable
	5	5	10		Stable
e	4	4	2	1.61	Partially Stable
	8	8	4		Partially Stable
f	2.5	2.5	5		Partially Stable
	5	5	10		Partially Stable

Oblate particles tend to have a single stable trapping site across all particle types and sizes considered. Their shape profiles resemble that of ordinary lenses, focusing light to a single spot. There is a single position that minimizes the divergence of light exiting the particle. However, oblate spheroids are also unstable because they are likely to tip to one side. The degree to which they will tip cannot be predicted, as it greatly varies from particle to particle. In contrast, prolate particles have multiple potential trapping sites along an aberrated beam. This arises due to an interplay between the phase shifts in the incident beam, which cause aberration, and the high-refractive power of the prolate shape particles. Consequently, several shallow optical traps appear. Based on our data in Fig. 7, we conclude that flatter, more disk-like particles are less likely to be stably trapped overall. The multiple traps exhibited for needle-like particles suggest that their most likely escape route involves a series of hops between shallow traps, induced by Brownian motion and torques.

Our simulations show a general adherence to optical trapping theory for different particle materials—materials with refractive indices slightly larger than the suspending medium tend to be trapped by optical gradient forces. This explains the trapping stability of PP particles, which have a low refractive index and are stably trapped by optical gradient forces. Both HDPE and PET particles have significantly higher refractive indices than PP particles. The trap depth for HDPE particles is about half that for PP particles, suggesting a higher rate of particle loss from the trap for HDPE. Large PET particles exhibit even less stability, as the restoring force regions are less frequent compared to smaller particles. The simulation results match our experimental observations, confirming the trends in optical trapping behavior.

## Conclusion

In conclusion, this work provides a greater understanding of microplastics’ optical trapping properties and how their characteristics change depending on material, color, size, shape, and laser wavelength. In particular, the study provides a better understanding of the limitations of using spherical models to predict the behavior of environmentally relevant, irregularly shaped microplastics. To investigate this behavior, we employed statistical analysis of 540 experimental observations of the optical trapping stability of different types of irregularly shaped microplastics, as well as numerical simulations of spheroidal particles with different sizes, shapes, and refractive indices. Since particles are trapped deeper inside the sample, spherical aberration reduces optical trap quality and particle stability. This effect is more pronounced for particles with a higher index of refraction, making optical trapping stability strongly dependent on the particle’s refractive index. Particle stability is also influenced by particle shape and size. Asymmetric particle shapes lead to unbalanced optical forces, inducing rotational motion or destabilization. Optical trapping is directly related to particle absorption; given the same materials, particles with higher absorption are less stable than those with lower absorption.

Among the tested materials, polypropylene (PP) in all colors and sizes exhibited the highest stability, likely due to its waterproof properties, low absorption, and optimal index of refraction. Polyethylene terephthalate (PET) particles larger than 10 μm exhibited the lowest stability due to their higher density and large index of refraction. NIR optical trapping minimizes heating and scattering effects while retaining high trapping stability, supporting its use for trapping microplastics in aqueous environments. The ability to optically manipulate irregular microplastic particles provides a foundational tool for controlled single-particle studies. This capability can be used to investigate microplastic interactions with biological cells, microorganisms, and environmental contaminants at the microscale. Such studies are crucial for improving the understanding of microplastic transport, bioaccumulation, and potential health impacts. In summary, this work demonstrates that optical trapping can serve as a powerful method for positioning and manipulating naturally weathered microplastics, enabling future environmental and biomedical research aimed at mitigating the impacts of plastic pollution.

## Materials and methods

### Ocean microplastic isolation

 Sand samples were collected from the wrack line of different zones of the Long Beach and Los Angeles area. If the samples were not processed within one hour of collection, they were stored in a refrigerator and processed within one week. A combination of density separation and sieving was used for microplastic isolation. First, a saturated NaCl solution (34 g of table salt per 100 mL of distilled water) was prepared. We used table salt for cost efficiency, as it provided a simple and effective medium for particle separation. The collected sample was spread in a steel tray, and the NaCl solution was poured over it and mixed for 5 min to separate organic and plastic particles from the sand. The mixture was filtered through a 220 μm sieve into a beaker, allowing smaller microplastics to pass through. The solution was left to settle for 1 h in the steel tray and an additional hour in the beaker, allowing particles to separate based on density. Microplastics, being less dense than the NaCl solution, floated to the surface and were collected into 1 mL tubes. To further purify the samples, the samples were centrifuged at 17 G for 5 min, causing microplastics to rise to the top of the test tubes. The isolated particles were collected from the surface and observed under a microscope. Raman spectroscopy analysis indicated that most of the identified microplastic particles were PP, often containing some contaminants.

### Sample preparation for absorption spectra measurements

 To prepare microplastic samples, selected plastic items were sanded down using fine sandpaper. During this process, it was observed that small fragments of sandpaper were mixed with the microplastics. As a result, it is assumed that there is a 10% error in the absorption spectra measurements, which represents an upper-bound estimation of contamination. The sanded plastic was stored in microtubes. Since microplastics tend to float on the water’s surface, absorption measurements were conducted in soybean oil, with respect to the oil baseline. To obtain the absorption spectra, 3.6 mg of lab-made microplastics were suspended in 3 mL of soybean oil, and the prepared sample was then placed into a cuvette and inserted into a spectrophotometer for measurement.

### Sample preparation for optical tweezers measurements

 For optical tweezer experiments, metallic files were used to produce lab-made microplastic samples in order to avoid contamination. A separate stainless-steel file was employed for each type of plastic sample, ensuring that there was no cross-contamination between the lab-made microplastics during optical studies. The metallic file method reduces contamination compared to using sandpaper, but it requires significantly more time for sample preparation. For instance, preparing a microplastic sample for absorption measurements takes about 1 h using fine sandpaper. In contrast, preparing the same amount of microplastic with a metallic file requires 5 to 10 h per sample. For optical trapping experiments, fresh samples were prepared by diluting microplastics in DI water with a small amount of surfactant. After 10 to 20 min of observation, new slides were used to minimize the floating effect and water absorption caused by PET microplastics.

### Optical tweezers experimental setup

 The arrangement of optical components in the setup is shown in Fig. 3. The single-trap optical tweezers system is built around an inverted microscope (Olympus IX83), with an optical breadboard for the components located just before the microscope objective. The experimental setup includes two continuous-wave, linearly polarized lasers: a blue 473 nm laser (Laser Quantum, gem 473 DPSS) and a tunable NIR Ti: sapphire laser (Msquared, SolsTiS 4000 PSX XF) with selected wavelengths 780 nm and 820 nm. Reflective collimators (Thorlabs RC08FC) are used to direct the light into a single-mode fiber, which then directs the light into the microscope. The advantage of incorporating collimators and fiber is that it simplifies the alignment of laser beams of different wavelengths into the microscope. A high numerical aperture objective (Olympus 1.25 NA 100X) focuses the laser beam on the sample, creating a single optical trap for holding and manipulating micro-sized objects. Once the microplastic is trapped, it can be held stationary while the microscope stage is moved using the MicroStage (Mad City Labs). All tests were conducted with 40–50 mW of laser power at the focal plane, and the trapped particles were positioned at a depth of approximately 10–50 μm from the cover glass. A camera (Hamamatsu, ORCA-FLASH4.0LT), with a dichroic mirror and notch filter in front of it, was used to image the particles.

### Raman spectroscopy

 Raman spectra of microplastics were obtained using a Raman spectrometer (Ocean Insight, MAYA2000PRO-RAMAN) attached to an inverted Olympus microscope. A 532 nm green laser was used as the excitation source, illuminating the sample through a 40X objective with a laser intensity of 3–5 mW before the objective. Each spectrum of the dried microplastic sample was collected with a data update rate of 5 ms, an integration time of ~ 1.5 s, ~ 7 scans averaged, and a boxcar width of ~ 3. Ten spectra were analyzed and averaged for each sample. To minimize contamination, plastic samples were thoroughly cleaned with 70% ethanol before being ground down using different stainless steel nail files.

### Modeling theory of light interaction

 A combination of two computational packages was used to model optical forces and torques on particles^32,42^. These packages solve the boundary value problem of electromagnetic scattering using a partial wave expansion of vector wavefunctions that satisfy the vector Helmholtz equation. Two sets of sizes and shapes were considered for the simulations: prolate spheroids with semi-axis diameters of 2.5 μm × 2.5 μm × 5 μm for small particles and 5 μm × 5 μm × 10 μm for large particles, and oblate spheroids with semi-axis diameters 4 μm × 4 μm × 2 μm for small particles or 8 μm × 8 μm × 4 μm for large particles. Since spheroids have larger volumes for the same axis lengths, for numerical simulation smaller sets of sizes were chosen to represent the particles. The simulation of 10 μm oblate particles is unreliable due to limitations of the calculation method, so 8 μm oblates were used as large particles. The refractive index of the particles in the simulation was chosen at the middle of the range for each type of material. In the calculations of momentum transfer per light quantum, the trapping light was assumed to have a wavelength of 780 nm, and transmitted 10 microns into the sample using a high-numerical aperture objective with NA = 1.25, matching the experimental conditions.

## Electronic supplementary material

Below is the link to the electronic supplementary material.

Supplementary Material 1

Supplementary Material 2

Supplementary Material 3

Supplementary Material 4

Supplementary Material 5

Supplementary Material 6

Supplementary Material 7

Supplementary Material 8

Supplementary Material 9

## Data Availability

All data generated or analyzed during this study are included in this publication. The additional images are available from the corresponding author upon reasonable request.
